# Mental Health of Cystic Fibrosis Patients and the COVID-19 Pandemic in Poland: A Single-Center Comparative Study

**DOI:** 10.3390/ijerph192316056

**Published:** 2022-11-30

**Authors:** Magdalena Humaj-Grysztar, Marta Rachel, Olga Śmiech-Michalec, Joanna Bonior

**Affiliations:** 1 Laboratory of Fundamentals in Midwifery Care, Institute of Nursing and Midwifery, Faculty of Health Sciences, Jagiellonian University Medical College, 31-501 Cracow, Poland; 2 Institue of Medical Sciences, Medical College of Rzeszow University, 35-310 Rzeszow, Poland; 3 Allergology Outpatient Department, Provincial Hospital No. 2, Lwowska 60, 35-301 Rzeszow, Poland; 4 Department of Medical Physiology, Chair of Biomedical Sciences, Institute of Physiotherapy, Faculty of Health Sciences, Jagiellonian University Medical College, 31-126 Cracow, Poland

**Keywords:** cystic fibrosis, mental health, depression, COVID-19, pandemic

## Abstract

Research shows that people with cystic fibrosis are more prone to suffer from psychological problems than healthy people; thus, the outbreak of the COVID-19 pandemic in Poland could have had an impact on their mental health. To assess this impact, we examined the mental health of patients before and during the pandemic. Survey participants were asked to fill in questionnaires that consisted of Beck Depression Inventory (BDI), 12-Item General Health Questionnaire (GHQ-12) and Cystic Fibrosis Questionnaire-Revised (CFQ-R; for the purpose of the study, an emotional functioning domain was used) during their hospital visits. A total of 81 patients took part in the study: 39 before the COVID-19 pandemic (BP) and 42 during the COVID-19 pandemic (DP). Patients’ medians were lower for the BDI, GHQ-12 and higher for the emotional domain of CFQ-R during the pandemic (3, 6, 75 vs. 4, 10, 73.33). Fewer patients felt that their mental health had deteriorated during the pandemic (Δχ^2^ = 7.723; *p* = 0.005), and GHQ-12 scores were lower in the DP group (Z = −3.044; *p* = 0.002). No significant differences were found between groups in terms of experiencing depressive symptoms (Δχ^2^ = 1.036; *p* = 0.309). It was found that patients with cystic fibrosis from our study group not only maintained but also improved their mental health state during the COVID-19 pandemic.

## 1. Introduction

### 1.1. Cystic Fibrosis

Cystic Fibrosis (CF) is the most common genetically determined disease among Caucasians. It is inherited in an autosomal recessive manner [1]. According to the European Cystic Fibrosis Society Patient Registry annual report from the year 2020, there were 52,246 registered CF patients living in 39 of the European and neighboring countries. Adults accounted for 53.1% of the total number of patients. In Poland, 1341 individuals with CF were included in the report, with 33.26% of them being adults [2]. The cause of this illness is CFRT gene mutation which is the most common mutation (deletion of three bases leading to a lack of phenylalanine at position 508), which results in disturbances in CFTR protein maturation. This leads to an impairment of the transport of chloride ions from the cells and an increased absorption of sodium ions, which results in reductions in water content in exocrine gland secretions, manifested by their thickening and increased adhesiveness. Due to the residual thick discharge, it is common for CF patients to suffer from the *Pseudomonas aeruginosa* pulmonary infections and develop the exocrine gland duct obstruction, which can lead to chronic inflammation. Although respiratory problems are the best-known symptoms, CF is a systemic disease in which the functioning of the pancreas, intestines, salivary, sweat glands and the reproductive system is impaired [1].

### 1.2. Cystic Fibrosis and Mental Health

Due to the chronic nature of the disease, the gradually deteriorating health condition and the inconvenience of therapy CF mean both children and adults experience anxiety and depression more often than the healthy population [3,4,5,6]. Depressive episodes are a known factor influencing the deterioration in perceived health and treatment in chronically ill patients; thus, it is important to consider mental health issues as well as physical ones [4,7]. As stated by the European Cystic Fibrosis Society, every CF patient aged 12 and above should undergo a psychological assessment by a psychologist who is a member of the CF multidisciplinary team [8]. For children aged 7–11, International Committee on Mental Health in Cystic Fibrosis recommends clinically evaluation for depression and anxiety in case of observed depression or anxiety symptoms or increased scores for depression or anxiety in their caregivers [9]. According to the Polish Cystic Fibrosis Society in the year 2019, 3 of the 13 polish Cystic Fibrosis Treatment Centers did not have a psychologist in their multidisciplinary teams [10]. According to a study by Cronly et al., depression and mental health have a greater impact on CF patients’ health-related quality of life than physical factors, thus proving the importance of mental health assessments as an element of patients’ care [11]. Although studies show a general trend of a low perceived quality of life amongst people with CF, not every aspect is negatively affected [12,13,14]. Studies by Uchmanowicz et al. conducted among polish CF patients revealed that, although surveyed general quality of life was low, their emotional functioning domain and mental health was above average [13,14]. In another study, Cronly et al. assessed positive mental health and wellbeing in people dealing with CF, which resulted in high scores for both the measured variables [15]. According to the systematic review of factors associated with health-related quality of life by Habib et al., FEV1% and pulmonary exacerbations had the strongest impact, and BMI and age were also correlated with the quality of life [16].

### 1.3. COVID-19 in Poland

The first case of COVID-19 in Poland was detected on 4 March 2020, which initiated a number of restrictions, such as the closure of educational institutions and state borders [17]. The state of the epidemic was announced on 20 March and, on 24 March, further restrictions were imposed on gatherings, meetings and events, leaving home beyond essential situations, and the use of public transport [18]. On 31 March, restrictions were introduced in everyday functioning to limit the spread of the virus, such as: limits to the amount of people occupying a store—up to three people per cash register—the obligation to maintain a minimum distance of 2 m between pedestrians, prohibition of using parks, or closing of hairdressing salons and suspension of rehabilitation treatments. Medical rehabilitation centers were not opened until 4 May, in the second stage of lifting restrictions. Although restrictions were gradually eased over time, social distancing was still recommended. Wearing masks (outside, and then only indoors), the widespread availability of disinfectants and the obligation to use them, maintaining social distancing, and limiting the presence of people with symptoms of infection in common rooms have formed the new everyday life routine. The first COVID-19 vaccines appeared on 27 December 2020, more than 9 months after the first case was diagnosed in the country [19]. CF patients were encouraged to take the vaccines by the polish CF societies [20].

COVID-19 restrictions, fear of contracting the virus, difficulties in accessing medical care and rehabilitation have become the new reality for every Polish citizen. The sudden need to stay at home and study or work remotely has disrupted the daily lives of many people. In a study conducted by Zhang et al. at the beginning of COVID-19 pandemic, focusing on the population of people living in China, increased levels of stress were reported due to the ongoing pandemic. Despite the increased level of stress, respondents stated that, at that time, they paid more attention to their mental health and received more family and social support [21]. Studies shows that the COVID-19 pandemic negatively affected the mental health of non-infectious chronic disease patients and people from the affected countries [22,23]. A Greek study conducted by Louvardi et al. found significantly higher levels of distress in chronic disease patients and in the selected group of respiratory disease patients compared to healthy individuals during the pandemic. Although the elevated levels of distress were significant, there were no differences in incidences of depression amongst the studied groups [24]. Similar results were obtained in the study by Graziano et al., where CF patients’ perceived level of stress one year after the onset of the COVID-19 pandemic was rated as moderate; however, their anxiety and depression scores were similar to those before the pandemic [25]. As CF patients have already dealt with the burden of their disease, the new challenges and threats of the pandemic may have contributed to the deterioration of their mental health. However, previous experiences of self-isolation and maintaining a strict hygiene regime in connection with the disease have a protective effect on their psychological well-being. The aim of this study was to assess and compare the mental health of CF patients before and during the COVID-19 pandemic in Poland.

## 2. Materials and Methods

### 2.1. Study Collection

The cross-sectional study was carried out in 2019 and 2020 at the Department of Allergology and Cystic Fibrosis and the Cystic Fibrosis Outpatient Clinic operating in the Provincial Clinical Hospital in Rzeszow, Poland. The time of data collection was divided into two timeframes: before the pandemic (from 2019 to 31 March 2020) and during the COVID-19 pandemic (from 31 March to 31 December 2020). Study participants were CF patients who were recruited during routine checkups at the clinic and during hospitalization due to bronchopulmonary exacerbations. All the participants were informed about the anonymity of the survey and its objectives. They were also informed that they could withdraw their consent at any time. After obtaining their consent (in the case of underage patients, their legal guardians were also asked for consent) they were asked to fill in the study questionnaire. Every questionnaire from underage patients was collected in the presence of a parent or legal guardian and a hospital psychiatrist to ensure that each question was clear and understandable. The study was approved by the Bioethics Committee of the Jagiellonian University Medical College, approval number 1072.6120.191.2018.

### 2.2. Measurements

Every questionnaire consisted of questions considering the socio-economic data of the respondents such as age, level of education or place of residence. To assess their mental health and well-being, three questionnaires were applied: Beck Depression Inventory, 12-Item General Health Questionnaire and Cystic Fibrosis Questionnaire-Revised.

The Beck Depression Inventory (BDI) is a tool used to assess the intensity of the depression symptoms in the surveyed population. It consisted of 21 questions regarding the depressive symptoms, every question had four answers, which were scored on a scale from 0 to 3 points. Respondents were asked to choose the answer that describes their feelings in the most accurate way during the previously set time period (last day, week or month). The scale ranges from 0 to 63 points, which were categorized as follows:

- 0–11 points—no depression symptoms;
- 12–26 points—mild depression symptoms;
- 27–49 points—moderate depression symptoms;
- 50–63 points—severe depression symptoms [26].

The 12-Item General Health Questionnaire (GHQ-12) is a tool used to assess mental health state. The questionnaire consists of 12 questions regarding mental health state, with four possible answers: less than usual, no more than usual, rather more than usual, much more than usual. Respondents are asked to pick the answers that best suit their feelings over the past few weeks. The answers are scored from 0 to 3 points depending on the given answer, with maximum score of 36 points. For the purpose of the study, the results were categorized as follows:

- 0–9 points—no mental health deterioration;
- 10–18 points—mild mental health deterioration;
- 19–27 points—moderate mental health deterioration;
- 28–36 points—severe mental health deterioration [27].

Cystic Fibrosis Questionnaire-Revised (CFQ-R) is a tool used specifically to assess CF patients’ quality of life. There are three different questionnaires, depending on the age of the respondents: CFQ-R14+ for teenagers aged 14 and older, CFQ-R 12–13 for teenagers aged 12–13 and CFQ-R 6–11 for children from 6 to 11 years old. CFQ-R14+ consists of 50 questions that are divided into nine quality of life domains, such as: physical, role/school, vitality, emotion, social, body image, eating, treatment burden, health perceptions and 3 symptom scale: weight, respiratory, and digestion. The other two questionnaires do not have vitality, health, role and weight domains. Questions can be considered as negative and positive, and each of them have four answers based on a 4-point Likert scale. After calculating the results for each domain, which can range from 0 to 100 points, the higher the score, the better the quality of life in that domain. For the purpose of the study, emotional functioning domain was used [28].

The data obtained in the study were divided into two groups: before the COVID-19 pandemic (BP) and during the COVID-19 pandemic (DP).

### 2.3. Data Analysis Method

Data were analyzed using Statistica.13. For the descriptive variables descriptive statistics such as percentage, mean, median, standard deviation, minimum and maximum were used. To assess the normality of the data distribution Shapiro–Wilk test was performed. For the data that did not present normal distribution and for those presented on the nominal scale, non-parametric tests were used. To assess the correlation between the variables in both groups, Spearman’s correlation was carried out. Mann–Whitney U test was performed to check the difference between the BP and DP group in the obtained values in BDI, GHQ-12 and CFQ-R in the emotional domain. Pearson’s chi-squared test was used to compare the incidence of depression and deterioration in mental health depending on the state of the pandemic. The significance level for the study was set at α = 0.05.

## 3. Results

### 3.1. Sample Characteristics

All participants were CF patients aged 6–45 years old. A total of 81 patients were recruited to the study. The BP group consisted of 39 participants with the mean age of over 21 and DP—42 with the mean age of 19. The majority of the respondents from both groups were adults, single and village residents (BP 72%, 79%, 69%; DP 64%, 93%, 71%). Over half of the before-the-pandemic group consisted of females (54%), with the opposite situation in the second group, where males accounted for 60%. According to Global Initiative for Chronic Obstructive Lung Disease (GOLD) classification of airflow limitation severity, approximately half of every group presented with a mild level of pulmonary obstruction, and very severe obstruction was more common in the DP group (12%) than in BP group (10%) [29]. To assess whether the two study groups differed in terms of the described sociodemographic variables, Pearson’s chi-squared test was performed and showed no statistically significant differences (Table 1).

### 3.2. Descriptive Statistics

Respondents scores were presented in Table 2. An additional Shapiro–Wilk test was performed, resulting in normal distribution only in CFQ-R emotional functioning domain scores in DP group (*p* = 0.094). Thus, results will be described using medians instead of means, and non-parametric tests are used. Beck Depression Inventory median was 4 for the BP and 3 for DP group (SD 8.485 vs. 5.885), with a higher maximum score for the BP group (29 vs. 22). A similar trend was found in 12-Item General Health Questionnaire. The before-the-pandemic group presented higher median (10 vs. 6) and maximum scores (27 vs. 20). CFQ-R emotional functioning domain and minimum scores were lower in the BP group (73.33 vs. 75; 0 vs. 33.33).

### 3.3. Depression Symptoms and Mental Health Deterioration Prevalence

The majority of respondents did not experience any depression symptoms before or during the pandemic (76.92% vs. 85.71%; Δχ^2^ = 1.036, *p* = 0.309). However, more than half of the patients before the pandemic showed any mental health deterioration incidence (53.85%) and only 23.81% during the COVID-19 pandemic. The results were statically significant (Δχ^2^ = 7.723, *p* = 0.005), as shown in Table 3.

### 3.4. Depression and Mental Health Deterioration Levels

The study assessed respondents’ levels of depression symptoms and mental health deterioration episodes as none, mild, moderate and severe. A detailed list of the obtained results in the form of numbers and percentages is presented in Table 4.

**Figure 1:**
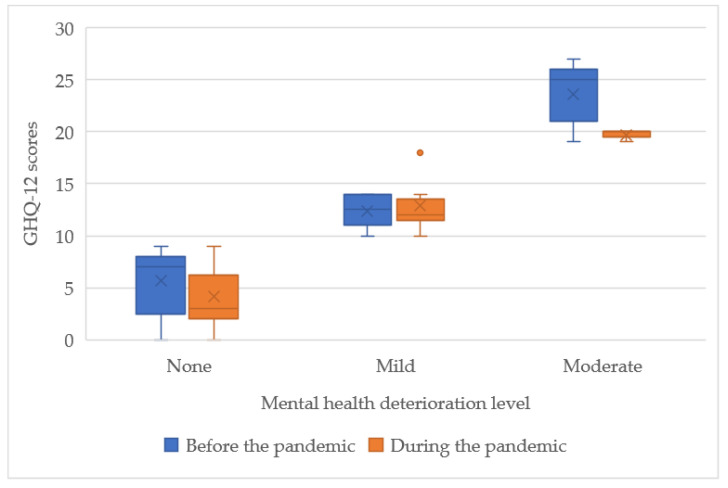
Interquartile range of the BDI scores of the studied groups.

Mild depression symptoms were found in six patients form both groups (BP 15.38% vs. DP 14.29%). As presented on the box-plot in Figure 1, during the pandemic, group scores were less scattered compared to those obtained before the pandemic. However, the DP group had higher median obtained scores (2.5 vs. 2 and 17 vs. 15.5). Only patients before the COVID-19 pandemic showed moderate depression symptoms (7.69%) in the range of 28–29 points. None of the surveyed participants experienced severe depression symptoms.

**Figure 2:**
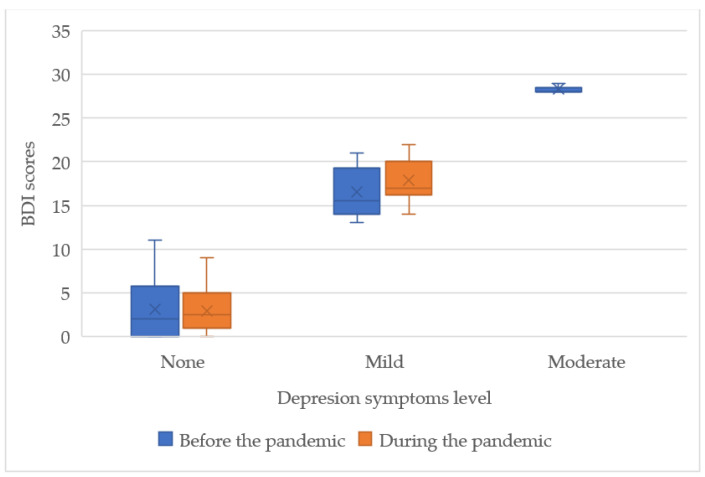
Interquartile range of the GHQ-12 scores of the studied groups.

As shown in Figure 2, more varied results were seen in the mental health deterioration levels, where mild episodes were found in 41.03% of patients before the pandemic, and only 16.67% in patients during the pandemic. Contrary to depression, moderate deterioration was observed in both groups. GHQ-12 scores of patients during the pandemic were much lower and of a lower range, with 19–20 points. A higher percentage of patients experienced mild mental health deterioration before the pandemic (12.82% vs. 7.14%), although, the highest score in the mild level was observed in the DP group as an outlier of 18 points. Similarly to depression symptoms, severe episodes were not observed and DP group scores were less scattered.

### 3.5. Beck Depression Inventory, 12-Item General Health Questionnaire and Cystic Fibrosis Questionnaire-Revised Emotional Domain Scores

The Mann–Whitney U test was carried out to see if there was a difference between the two research groups in terms of the results obtained in the used research tools. The Beck Depression Inventory test showed no statistical significance (Z = −0.586; *p* = 0.558). On the other hand, a 12-Item General Health Questionnaire scores statistically differed between the groups (Z = −3.044; *p* = 0.002). Patients during the pandemic had lower scores in the GHQ-12, which means they perceived their mental health as being better than those in the before-the-pandemic group. Results from the Cystic Fibrosis Questionnaire-Revised emotional domain scores did not differ amongst groups (Z = 1.011; *p* = 0.312).

### 3.6. Beck Depression Inventory, 12-Item General Health Questionnaire and Cystic Fibrosis Questionnaire-Revised Correlations

BDI was positively correlated with GHQ-12 results (BP r = 0.664; DP r = 0.468) and negatively with the emotional functioning domain of CFQ-R (BP r = −0.645; DP r = −0.491) indicating that with the increasing intensity of depression regarding the mental health of the respondents was deteriorating, and the emotional domain of the CFQ-R values was decreasing. A negative correlation was found between GHQ-12 results and emotional functioning (BP r = −0.747; DP r = −0.480). The age and Body Mass Index of participants were also checked for possible correlations with used scales. Age was positively correlated with BDI in the before-the-pandemic group (r = 0.399) and GHQ-12 in both groups (BP r = 0.360, DP r = 0.338), showing that the higher the age of the patients, the depression symptoms were more severe and their mental health was deteriorating. BMI only showed a correlation with GHQ-12 results during the pandemic (r = −0.446), indicating that the higher the BMI, the lower the deterioration in mental health in the study group. All the correlations were statistically significant (*p* < 0.05). There were no statistically significant correlations between saturation, FEV1% and used scales in any of the studied groups. On the other hand, FEV1% had a moderate negative correlation with age (BP r = −0.598; DP −0.543) and a moderate positive with saturation (BP r = 0.603; DP r = 0.663) at *p* < 0.05.

## 4. Discussion

The presented study investigated and compared the mental health of cystic fibrosis patients before and during the COVID-19 pandemic. 

In this study, we assessed the occurrence of depression symptoms and their severity. However, the during-the-pandemic group had fewer incidences of depression symptoms (14.29% vs. 23.08%), although the difference was not statistically significant, which indicates that COVID-19 pandemic did not influence depression in the studied group of CF patients. Similar results were shown in an Italian study by Ciprandi et al., where 90% of participants did not present any depression symptoms during the pandemic [30]. Research from the United Kingdom also confirmed low rates of depression and did not identify significant changes in those rates during the pandemic [31]. Those results differ from that of Graziano et al., where 75% of the study participants manifested symptoms of depression [32]. In another study by Graziano et al., 45% of CF adolescents and adults scored above the depression cut-off one year after the beginning of the pandemic, which was similar to the results of that population prior to the pandemic [25]. Although the frequency of depression was higher than in our study, the general tendency was similar—COVID-19 did not influence the occurrence of depression in CF patients. A slight increase was reported by the small sample study by Rhoads et al., from 8.9% of respondents showing depression symptoms in 2019 to 10% in 2020 [33]. A New-York-based study also presented elevated levels of depression in CF patients during the COVID-19 pandemic, from 38% to 45%, but the results were statistically insignificant [34]. In our study, the amount of mild depression symptoms was in both groups, with 15.38% before and 14.29% during the pandemic, but moderate symptoms were only present in the before group (7.69%). Smith et al. found moderate to severe depression symptoms in 12% of CF patients while, in our study, there was no incidence of that level during the pandemic [35]. In Ciprandi et al.’s research, 8.6% of CF patients presented mild to moderate level of symptoms and 1.4% presented severe symptoms [30]. This may indicate that Polish patients with CF during the COVID-19 pandemic were less susceptible to the development of moderate to severe depression symptoms.

In the current study, the mental health state of survey participants was assessed. The mean, median and the maximum score of GHQ-12 were higher before the pandemic (10.718, 10, 27 vs. 6.738, 6, 20). There was also a statistically significantly higher percentage of patients showing any deterioration in mental health (53.85% vs. 23.81%; *p* = 0.005). None of the respondents suffered from severe mental health deteriorations, but fewer mild and moderate incidences were found during the pandemic. These results show that, despite the difficulties of the global COVID-19 pandemic and national restrictions, the mental health of CF patients has not deteriorated; furthermore, it has improved. These results are quite surprising, as many studies about CF patients during the pandemic presented the opposite results. From the beginning of the COVID-19 pandemic in Poland, the CF foundations was very active in providing support, including psychological support, to patients. Combined with the increased frequency of contact with medical staff through online consultations, this could have contributed to increasing the sense of security among patients and reducing anxiety.

Elevated levels of distress were present amongst Belgian CF patients [36]. Westcott et al. found that the incidence of anxiety in patients increased from 27% to 54% during the pandemic, similar to results presented by Rhoads et al., where the prevalence of anxiety increased in adult CF patients during the pandemic from 25% to 33.3% [31,33]. In small sample Italian study, only 25% of adolescents and young adults with CF were free from anxiety [32]. A Chinese study by Liang et al. showed that 40.4% of the surveyed young CF patients were prone to psychological problems. Additionally, 14.4% were diagnosed with post-traumatic stress disorder [37]. Statistically significantly increased incidences of anxiety amongst CF patients during the pandemic was also shown in a study by Simonson et al. [34].

According to Collaço et al., over 75% of studied CF patients worried about how possible COVID-19 infection might influence their health [38]. In the German study about COVID-19-related fears, people with CF assessed the risks of the pandemic, severe course of the disease and the possibility of death due to COVID-19 infection as being more serious than they were for than healthy people [39]. The severity of lung disease was the factor causing the highest levels of worry about the possibility of health deteriorations during the pandemic in the study by Radtke et al. [40]. This fact may be important in the context of disease progression in people with CF and may suggest which groups of patients should receive special psychological care. During the pandemic, patients from our study group were treated with telemedicine solutions in the form of an electronic stethoscope, which allowed for patients to be auscultated at home. Patients sent the results of these self-examinations to their doctors and nurses on a daily basis. Daily contact with the medical team and ongoing health assessments could reduce patients’ concerns about their health. This could have contributed to improvements in their mental state, despite the ongoing pandemic. 

Despite the general trend of mental health deteriorations, some research shows similar results to ours. An Italian study by Ciprandi et al. that surveyed and compared CF patients and the general population found that distress levels were elevated but similar in both groups, but slightly higher in the control group [30]. Studies suggest that the mental health of children with CF were less affected by the pandemic. Yanaz et al. showed that CF patients aged from 7 to 18 years old were less anxious about the COVID-19 pandemic and, in general, were more resilient compared with their healthy peers [41]. A Turkish study by Senkalfa et al. revealed that CF children had lower levels of anxiety than their healthy peers; however, their mothers and the mothers of healthy children reported higher levels of anxiety during the pandemic [42]. Beşer et al. found statistically significant lower anxiety prevalence in children with CF than amongst healthy children [43]. These results are similar to ours, and we found that age was positively correlated with GHQ-12 scores, indicating that the mental health of people with CF deteriorates with age. The study by Güre et al., conducted among people with rare diseases, found that age was positively correlated with the fear of contracting the COVID-19 virus [44]. The lower levels of distress and anxiety in children and adolescents with CF during the pandemic may have been due to the fact that, prior to the pandemic, young patients were used to maintaining good hygiene, often had to stay at home for health reasons and were often home-educated. Considering the above, it could be concluded that their lives did not significantly change as a result of the outbreak of the COVID-19 pandemic.

In our study we assessed CF patients’ quality of life in the emotional functioning domain of CFQ-R. In both groups, the mean and median of obtained scores were above 50, suggesting good quality of life in the assessed domain. The pandemic group had higher mean, median and minimum scores (72.619, 75, 33.33 vs. 66.623, 73.33, 0); however, the Mann–Whitney U test did not confirm statistically significantly higher scores in the during-the-pandemic group (Z = 1.011; *p* = 0.312). No statistically significant changes before and during the COVID-19 pandemic were found in the study based in Los Angeles, USA [45]. Studies conducted prior to the pandemic showed a good quality of life in the emotional domain in people with CF. A study by Tomaszek et al. presented similar results regarding emotional functioning scores (median 77) in Polish 14-to-25-year-old CF patients [46]. A high mean of emotional functioning score (75.3) was also confirmed in Brazilian patients. Similar to our study, there was no statistically significant correlation with FEV1%. However, BMI was negatively correlated with the emotional functioning domain (r = −0.31, *p* ≤ 0.05) [47]. In the French study, adults with CF had the highest median (80) in the emotional domain [48]. Considering these results, we concluded that the COVID-19 pandemic did not influence the perceived quality of life in the emotional functioning domain of our study group. 

Although the COVID-19 pandemic was a challenging time for people with CF, it also resulted in some positive effects and conclusions. One of them was raising awareness about the necessity of mental-health-monitoring and promotion amongst not only the patients but also their caregivers and therapeutic teams [35,49]. Although studies show that, during lockdowns, the physical activity of patients was often disturbed and less frequent, it also caused more consistency in their daily respiratory therapies and more adherence to general therapies [36,40,50]. For comparison, in our study group, the frequency of home rehabilitation increased due to the pandemic, which could have a positive impact on the quality of life and well-being of patients.

## 5. Limitations of the Study

The authors of this study are aware of its limitations. Since it is a single-center study, the number of participants was limited and only a small percentage of people living in Poland with cystic fibrosis was included in the study. Every study questionnaire was collected in person, during respondants’ medical check-ups or during their hospitalizations. Since the outbreak of the COVID-19 pandemic, the number of patient visits to the hospital was limited to necessary visit, and contact with hospital staff was usually maintained via telehealth solutions. This was another reason why the number of surveys collected at that time was limited.

Despite the universal recommendations for CF centers in Poland, some differences in approaches to the patients, the lack of psychologist in the multidisciplinary CF teams and differentiation in the number of COVID-19 cases in different areas of Poland might influence the experiences of patients from other centers, and the obtained results may not be generalizable to the entire population of Polish CF patients. Future multi-center studies are needed to assess the general mental health of the studied group.

Due to the small number of survey participants, the scores of children, teenagers and adults were analyzed together, which might influence the final results. Additionally, the DP group consisted of a higher percentage of male patients (60% vs. 46%); however, the difference was statistically insignificant. As the study shows, the trend of poorer mental health in female individuals with cystic fibrosis, and the inequality between genders in our study, could have disrupted our findings [14,51,52].

The current study describes the mental health of cystic fibrosis patients from the first 9 months of COVID-19 in Poland. Due to the wider time range of data collection during the pandemic, patients could get used to this new reality and better deal with the consequences of the pandemic over time. For this reason, we could not interpret the results obtained as the initial response to the pandemic, but rather as a general picture of functioning during the early pandemic period.

## 6. Conclusions

To our knowledge, this study is the first to show the influence of the COVID-19 pandemic on mental health of CF patients in Poland. The COVID-19 pandemic did not increase the prevalence of depression symptoms in patients with CF in the studied group. Patients with CF during the pandemic felt fewer deteriorations in their mental health than those before the pandemic. The reason for such results might be due to the national COVID-19 restrictions, such as social distancing, the necessity of wearing masks in public places, the need for frequent hand disinfection, and remote working and learning, which might have created a safer environment for people with chronic illnesses such as CF. Rules and behaviors that, for healthy people, were new and inconvenient, such as maintaining hygienic restrictions and isolation, were part of everyday life for CF patients’, which might have been the reason that the COVID-19 pandemic did not negatively influence the mental health of the studied group. It is important to recognize the role of social support for patients during a pandemic and increase contact with medical staff through online consultations, which may turn out to be a good new practice in caring for people with CF.

## Figures and Tables

**Table 1:** Sociodemographic data of surveyed patients.

Variable		Before the Pandemic	During the Pandemic	*p* Value
		n (%)	n (%)	
Sex	Female	21 (54)	16 (38)	0.463
	Male	18 (46)	25 (60)	
Age	6–13	8 (21)	10 (24)	0.730
	14–17	3 (8)	5 (12)	
	18–45	28 (72)	27 (64)	
Marital status	Single	31 (79)	39 (93)	0.112
	Married	6 (15)	1 (2)	
	Divorced	2 (5)	2 (5)	
Educational level	In school	18 (46)	21 (50)	0.734
	High school	14 (36)	17 (40)	
	Vocational education	3 (8)	2 (5)	
	Higher education	4 (10)	2 (5)	
Employment status	Student	21 (54)	26 (62)	0.732
	Employed	4 (10)	2 (5)	
	Not employed	14 (36)	14 (33)	
Place of residence	Village	27 (69)	30 (71)	0.513
	City under 100,000 citizens	9 (23)	9 (21)	
	City over 100,000 citizens	3 (8)	3 (7)	
Pulmonary obstruction level by GOLD criteria	Mild	19 (49)	21 (50)	0.746
	Moderate	7 (18)	10 (24)	
	Severe	9 (23)	6 (14)	
	Very severe	4 (10)	5 (12)	
Health condition	Stable	16 (41)	17 (40)	0.960
	Exacerbation	23 (59)	25 (60)	

**Table 2:** Descriptive statistics of the scores obtained by patients and Shapiro–Wilk test results.

Variable		M	Me	SD	Min	Max	S-W
Beck Depression Inventory	Before the pandemic	7.103	4	8.485	0	29	*p* < 0.05
	During the pandemic	5.048	3	5.885	0	22	*p* < 0.05
GHQ-12	Before the pandemic	10.718	10	6.448	0	27	*p* < 0.05
	During the pandemic	6.738	6	5.575	0	20	*p* < 0.05
CFQ-R emotional functioning domain	Before the pandemic	66.623	73.33	23.24	0	100	*p* < 0.05
	During the pandemic	72.619	75	18.11	33.33	100	*p* = 0.09

**Table 3:** Prevalence of depression symptoms and mental health deteriorations.

Variable		Yes n (%)	No n (%)	*p* Value
Prevalence of depression symptoms	Before the pandemic	9 (23.08)	30 (76.92)	0.309
	During the pandemic	6 (14.29)	36 (85.71)	
Prevalence of mental health deterioration	Before the pandemic	21 (53.85)	18 (46.15)	0.005
	During the pandemic	10 (23.81)	32 (76.19)	

**Table 4:** Prevalence of depression symptoms and mental health deterioration levels.

Variable		None n (%)	Mild n (%)	Moderate n (%)	Severe n (%)
Depression symptoms level	Before the pandemic	30 (79.92)	6 (15.38)	3 (7.69)	0
	During the pandemic	36 (85.71)	6 (14.29)	0	0
Mental health deterioration level	Before the pandemic	18 (46.15)	16 (41.03)	5 (12.82)	0
	During the pandemic	32 (74.42)	7 (16.67)	3 (7.14)	0

## Data Availability

Data available on request due to restrictions privacy.
